# Not Just PA28γ: What We Know About the Role of PA28αβ in Carcinogenesis

**DOI:** 10.3390/biom15060880

**Published:** 2025-06-16

**Authors:** Paolo Cascio

**Affiliations:** Department of Veterinary Sciences, University of Turin, Largo P. Braccini 2, 10095 Grugliasco, Italy; paolo.cascio@unito.it

**Keywords:** PA28αβ, PSME1, PSME2, PA28γ, proteasome, proteasome activator, protein degradation, ATP-independent proteolysis, Ubiquitin Proteasome System (UPS), intrinsically disordered proteins (IDPs), MHC-class I antigen presentation, cancer, neoplastic transformation, cancer immune evasion

## Abstract

The ubiquitin-proteasome pathway performs a strictly controlled degradation of specific protein substrates within the eukaryotic cell. This catabolic mechanism allows the rapid removal of proteins damaged in any way, and therefore potentially capable of compromising cellular homeostasis, as well as the constant turnover of all cellular proteins, in order to balance their synthesis and thus maintain the correct levels of proteins required by the cell at any given time. Consequently, the ubiquitin-proteasome system plays a fundamental role in regulating essential cellular processes, such as the cell cycle, apoptosis, immune responses, and inflammation, whose dysregulation or malfunction can lead to neoplastic transformation. Not surprisingly, therefore, alterations in the activity and regulatory mechanisms of the proteasome are common not only in various types of tumors, but often represent a contributing cause of oncogenesis itself. Among proteasome modulators, PA28γ, due to its function in promoting cell growth and proliferation, while inhibiting apoptosis and cell-mediated immune responses, has received great attention in recent years for its well established pro-tumoral activity. Conversely, the role played in oncogenesis by the second paralogue of the PA28 family of proteasome activators, namely PA28αβ, is less clearly defined, which is also related to the lower level of general understanding of its cellular activities and biological functions. However, increasing experimental evidence has demonstrated that PA28αβ also plays a non-secondary role in the process of neoplastic transformation and tumor growth, both by virtue of its regulatory function on class I cell-mediated immune responses and through activity promoting cell duplication and growth. This review aims to summarize the current knowledge and evidence on the molecular mechanisms and cellular functions through which PA28αβ may support development and growth of cancer.

## 1. Introduction

In its canonical form, the degradative pathway of endogenous cellular proteins via the ubiquitin-proteasome system (UPS) involves their post-translational modification through covalent coupling with one or more polyubiquitin chains (i.e., polymers of the small globular protein ubiquitin, Ub), and subsequent proteolysis by the high molecular weight (2.4 MDa), multimeric 26S proteasome (a multicatalytic protease) in a process that requires energy provided by ATP hydrolysis. Structurally, the 26S proteasome can be further subdivided into a central cylindrical element, the 20S proteasome which directly carries out the proteolytic reaction in its inner cavity, and the 19S accessory particle made up of various multitask subunits including ATPases, receptors for the polyubiquitin chain, and deubiquitinases. In most organs and tissues, a constitutive 20S proteasome (20Sc) is expressed, which has six catalytic subunits that are capable of hydrolyzing peptide bonds of three different types: two β1 subunits, with caspase-like activity (i.e., cleavage after acidic amino acids), two β2 subunits, with trypsin-like activity (i.e., cleavage after basic amino acids), and two β5 subunits with chymotrypsin-like activity (i.e., cleavage after hydrophobic amino acids). However, in cells of hematological origin, and almost in all other cells of the body following stimulation with γ-interferon (INF-γ) or other pro-inflammatory cytokines (e.g., type-I interferons and TNF-α), the 20Sc proteasome is rapidly replaced by the immunoproteasome (20Si), an alternative form containing different proteolytic subunits (β1i, β2i, β5i) that are more efficient in generating the MHC class I epitopes [1]. The distinct elements of UPS catabolic pathway and their molecular structures and mechanisms of activity, its multiple and interdependent regulatory processes, and the biological consequences of alterations and impairments in its functions, have been the subject of many recent and in-depth reviews to which the reader is referred [2,3,4,5,6].

**Figure 1:**
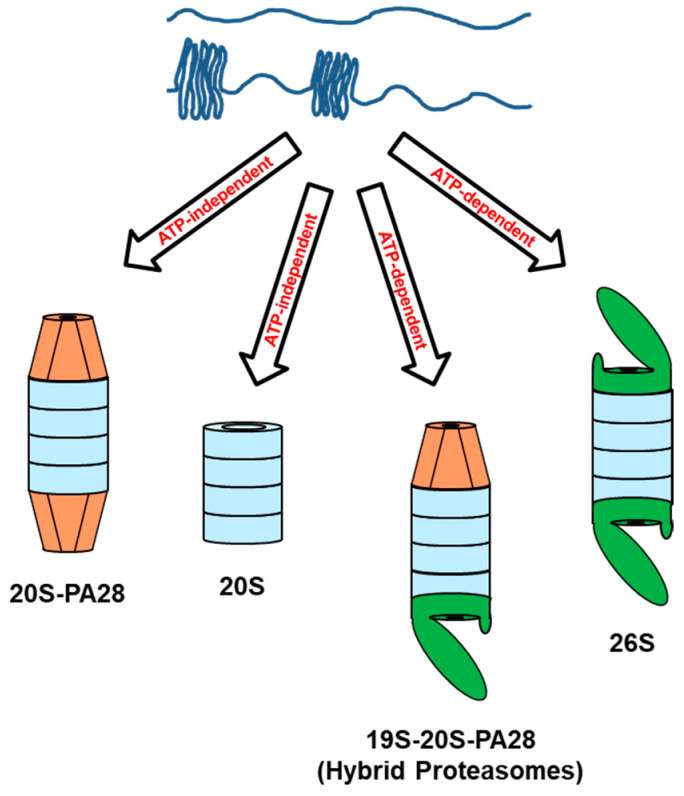
Ubiquitin-independent proteasomal degradation. Proteins that are completely or largely devoid of three-dimensional structure, either naturally (IDPs) or following chemical-physical damage or genetic aberrations, can be degraded by proteasomes in the absence of ubiquitin. This degradative pathway can take place with the 20S core particle (blue) alone or in association with activators such as PA28 (orange). In this case, the process does not require the energy provided by ATP. Alternatively, proteins devoid of tightly folded three-dimensional structure can also be degraded by the 26S proteasome in its canonical conformation (i.e., associated with one or two 19S caps, green) or in the form of a hybrid proteasome. In both these cases, proteolysis requires ATP. These degradation modes do not exclude a priori the possibility that a part of the destructured proteins can also be ubiquitinated and degraded by the 26S according to classical modes. It should also be emphasized that partial hydrolysis of only the unfolded portion of IDPs may be sufficient for their functional inactivation.

Interestingly, over the years there has been increasing experimental evidence indicating that alongside this canonical mechanism, which accounts for a significant part of the degradative activity carried out by the UPS in the cell, there are also variants or alternative pathways that hydrolyze proteins without the involvement of ubiquitin not only via the 26S proteasome holoenzyme but also by means of its proteolytic core 20S in the absence of 19S regulatory particles (Figure 1). In the latter case, the 20S particle can act either alone or in association with alternative modulators that, differently than the 19S particle, do not require ATP and Ub for their activity (see below). Specifically, the non-canonical forms of UPS based on the 20S core particle in the absence of 19S complex seem to be mainly involved in degrading proteins that, lacking a rigidly and tightly folded three-dimensional structure, can directly access the internal hydrolytic sites of the proteasome, thus avoiding the otherwise essential step of energy-dependent pre-unfolding [7].

In other words, its preferred substrates naturally belong to the category of intrinsically disordered proteins (IDPs) or alternatively have undergone denaturation following chemical-physical damages or genetic aberrations. Surprisingly, it has even been recently reported that the 20S proteasome can degrade polyubiquitin chains in vitro, although in this case it is unclear where the energy needed for the denaturation process that allows the substrate to enter the proteasomal proteolytic cavity comes from [7,8,9,10,11,12,13,14,15,16,17,18]. Molecular mechanisms and possible biological functions and relevance of these alternative UPS forms have been thoroughly discussed elsewhere [19,20,21,22,23].

### 1.1. UPS and Cancer

Since strictly controlled degradation of the proteome is a key factor in maintaining cellular homeostasis and allows a rapid and effective response to a variety of different stress challenges, it follows that perturbation of this delicate network is involved in the onset of various pathological conditions, including cancer [24]. Accordingly, alterations in the activity and regulation of proteasomes not only are a common feature of many tumors, but often represent a contributing cause of oncogenesis [25,26,27,28,29,30]. Furthermore, the possible role that malfunctions of the UPS could have in triggering and sustaining the neoplastic transformation of cells and tissues is also suggested by the crucial observation that small cell-permeable molecules that inhibit the proteolytic active sites of the proteasome display a clear and powerful tumoricidal activity in several cellular and animal experimental systems and models [31,32,33,34,35]. In line with this, some of these molecules are now commonly used as antitumor drugs, especially against hematological neoplasms such as multiple myeloma and mantle cell lymphoma. Moreover, new selective inhibitors of particular proteasome subpopulations (e.g., immunoproteasome or proteasome associated with specific activators) are also under study, which hopefully should present a more targeted and powerful activity against neoplastic tissues (especially solid tumors) while limiting unwanted harmful side effects on healthy tissues and organs [36,37,38,39,40,41,42].

### 1.2. The PA28 Family of Proteasome Activators

The members of the PA28 family (alternatively called 11S, REG, PMSE) of ATP- and Ub-independent proteasomal activators were identified and described independently by the laboratories of Rechsteiner and DeMartino in the early 1990s [43,44]. These initial biochemical studies and others shortly thereafter led to the characterization in mammalian cells of three highly homologous 28 kDa proteins, PA28α, PA28β and PA28γ, which are capable of associating with the 20S proteasome and strongly stimulating its ability to hydrolyze short peptides of 3–4 amino acids [45,46,47,48,49,50]. Of note, while PA28α and PA28β are expressed only in jawed vertebrates (with the notable exception of birds), PA28γ is present in jawed and jawless vertebrates and orthologs are also found in invertebrates and unicellular eukaryotes, such as Drosophila melanogaster, Rhipicephalus appendiculatus, Schistosoma mansoni, Dictyostelium discoideum, and Plasmodium falciparum [51,52,53,54,55,56]. In this regard, recent phylogenetic analyses indicate that PA28γ represents a slow-evolving gene (which probably retains the original functions of the ancestor precursor of the PA28 family) from which faster evolving sequences stemmed out, leading to PA28α and β, which are involved in acquired cell-mediated immunity [56]. Table 1 lists the main differences between PA28αβ and PA28γ. Of extreme interest, the degradation of full-length proteins (regardless of whether they were correctly folded, denatured, or ubiquitinated) was reported to be insensitive to the stimulatory action of PA28s. However, the degradation of proteins with different structural and chemical-physical properties was evaluated in detail only for PA28α and β, while for the third paralogue PA28γ it was only excluded that it could stimulate the hydrolysis of the native substrate lysozyme [57].

Many years later, an accurate, in-depth in vitro analysis of the degradative properties of PA28γ established its specific capacity to greatly stimulate the hydrolysis of unstructured proteins (i.e., devoid of a fixed or ordered three-dimensional structure) by the 20S proteasome in a process that does not require ATP and Ub [7]. This important finding provided a general interpretative model for the ability of PA28γ to enhance proteasomal degradation of some specific substrates [59,60,61,62,63], all of which appear to be proteins that are completely or largely devoid of a strongly folded arrangement [64].

Structurally, PA28 proteins associate with each other through both polar and hydrophobic interactions to form heptameric rings of about 210 kDA [65] that attach to the free ends of the 20S cylinder. In vivo, PA28α and β form a hybrid ring with a predominant stoichiometric composition of 3α4β or 4α3β [58,66,67,68,69]. In contrast, seven identical gamma subunits associate together to form a homoheptameric ring [70,71,72,73]. Both PA28αβ and PA28γ complexes can also associate with the free end of 26S proteasomes containing a single 19S to form so-called hybrid proteasomes whose biochemical properties and precise biological functions are currently unclear [64,74]. Electron and cryo-electron microscopy studies reveal a truncated cone-shaped structure of PA28αβ, with a base diameter of approximatively 10–11 nm (where it contacts the α-annulus of the 20S core particle) and a height of about 7–8 nm. Furthermore, PA28αβ is crossed by a longitudinal water channel of 2 nm diameter at the top and 3 nm at the base that aligns with the central pore of the proteasomal α-ring. In this way, a direct communication route is created that connects the internal proteolytic chamber of the proteasome with the outside [65,66,69,75]. This general organization of the quaternary structure has recently also been shown to apply for PA28γ homoheptamers, although in this case the diameter of the central aqueous channel appears slightly larger. This may at least in part explain its peculiar biochemical properties, and specifically its ability to stimulate proteolysis of unstructured substrates [73]. As expected for highly homologous proteins that exhibit a considerable degree of sequence identity [64], the secondary and tertiary organization of the monomers is very similar among the 3 paralogues, which all exhibit a compact globular structure, with 4 α-helices of 33–45 residues that are connected by 3 loops. Of particular importance is the loop located between α-helices 2 and 3 (the “activation loop”), which is largely responsible for the proteasome activation mechanism and the C-terminal tail of 10 amino acid residues that allows anchoring of PA28s to the proteasome [65,76,77,78,79,80].

A breakthrough in understanding the molecular mechanism of the stimulatory activity of PA28s was achieved in 2001, when the crystallographic structure of a trypanosome homologue of mammalian PA28 (i.e., PA26) assembled with 20S yeast was reported [81]. This important study revealed that the free C-terminal tails of the activator subunits, by inserting into small cavities present on the free surface of the proteasome α-annulus, provide the binding energy but are not directly responsible for the activation of the protease. Instead, activation is achieved through the highly homolog activation loops that interact with the N-terminal tails of proteasome α-subunits. In this way, the gate that seals the end of the free (i.e., not associated with any activators) 20S proteasome [82,83] is destructured. This results in the formation of an aqueous pore at the center of the α-ring, which allows the substrates to access, and the products to efflux out of the internal proteolytic chamber [84,85]. In more recent years, cryogenic electron microscopy studies of other PA28 complexes (e.g., PA28αβ or PfA28) in association with different 20S particles have confirmed this molecular mechanism of action, even if with slightly different modalities depending on the specific activators analyzed [55,69]. Moreover, in accord with previous biochemical analyses aimed at characterizing the molecular mechanisms of PA28 [64,74], these and others studies clearly indicate that, in addition to the main function of gate opening, the activators of the PA28 family also induce structural modifications that propagate from the ends to the internal catalytic sites of the protease, thereby altering its activity by an allosteric process [72,86,87,88,89]. Furthermore, the surface of the central channel that longitudinally crosses the heptamer is largely coated with charged amino acids, for both PA28αβ and PA28γ, which also suggests a selective filtering mechanism (i.e., based not only on size, but also on chemical-physical properties of the passing molecules) for the incoming substrates and outgoing products [18,64]. In this selective filtering mechanism, a specific role can also be played by the loops between α-helices 1 and 2 (homolog-specific inserts), which are arranged around the upper entrance of the PA28s and which constitute the most divergent portions between the three activators (i.e., α, β, and γ monomers) [58].

### 1.3. Biological Functions of PA28 Activators

As for the biological roles of PA28 activators, although they have been the subject of in-depth studies for more than 30 years and despite accurate structural (crystallography, cryo-EM, EM and structural MS) and biochemical studies, it must be recognized that an exact and comprehensive understanding of their functions has not yet been obtained [64,90]. PA28γ is a protein with a prevalent nuclear localization that is present in all tissues and organs (with particularly high levels in the spleen and brain) [48,91,92]. From early studies it was clear that it has a stimulatory activity on cell cycle progression and cellular proliferation and growth [93], while inhibiting apoptosis [94]. Subsequently, other functions have also been reported such as regulation of nuclear dynamics [95,96,97], preservation of centrosome and chromosomal stability [98], enhancement of cellular response to double-strand DNA breaks [99], perturbation of lipid metabolism as a consequence of impaired autophagy [100], stimulation of spermatozoa maturation [101,102], and blunting of MHC class I immuno responses [103,104,105]. Understanding the cellular and molecular mechanisms by which PA28γ achieves all these actions is further complicated by the finding that in vivo only an small fraction (<5%) of the protein is associated with the proteasome [106], which leaves open the possibility that part of its functions are also obtained in a protease-independent manner. Regardless of its precise molecular mechanism(s), however, there is no doubt that PA28γ, by virtue of its stimulating activity on cell proliferation and growth, angiogenesis, glycolysis, and epithelial-mesenchymal transition, as well as inhibition of apoptosis and MHC class I antigen presentation, represents a genuine oncoprotein whose levels are greatly increased in many types of tumors [64,107,108,109].

Even more uncertain, however, is the exact biological role of PA28αβ [74]. Since its discovery, PA28αβ has been considered to play a stimulating role in the process of MHC class I antigen presentation based on the consideration that, unlike PA28γ, it is greatly induced by γ-interferon (INF-γ) and highly expressed in lymphoid cells and organs [110]. Nonetheless, in vitro and in vivo studies aimed at clarifying its role in proteasomal antigen processing have shown more subtle and less generalizable effects than expected (see below). Additionally, a role of PA28αβ in the cellular response to oxidative stress has been hypothesized. In particular, it has been reported that PA28αβ is able to stimulate the degradation of oxidized proteins by the 20S proteasome [111,112,113]. However, other studies have yielded different results [114], and thus further investigations are necessary to definitively clarify this important aspect of PA28αβ function.

## 2. PA28αβ and Cancer

**Figure 2:**
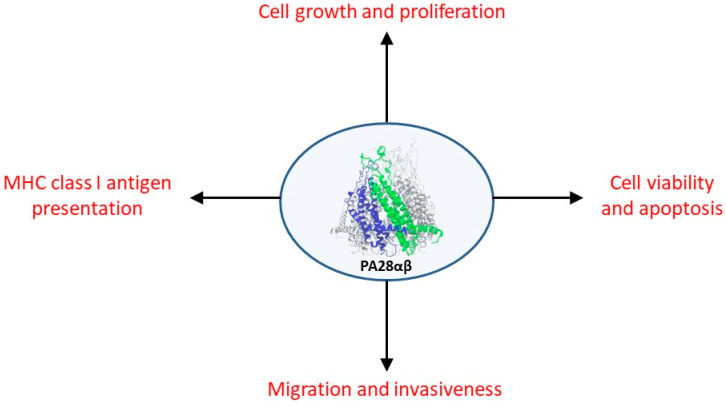
Biological processes that are affected by PA28αβ with a role in carcinogenesis and tumor spread. The precise function that PA28αβ plays in many of these pathways is not yet unequivocally determined and likely varies depending on the type of tumor (see text for further details).

Although many studies have clearly shown a relationship between alterations in the expression and activity of PA28αβ and the onset and development of various cancers in both humans and mice, the exact role(s) that this proteasomal activator plays in carcinogenesis is challenging to unequivocally establish and place within a general interpretative framework. This difficulty derives mainly from partially conflicting data reported in the literature regarding its role in tumor development and from the lack of a clear understanding of its molecular mechanisms of action and biological functions [74]. It is, however, possible to identify different functions of PA28αβ related to tumorigenesis, both in light of its role in MHC class I antigen presentation (alterations in which are likely to facilitate tumor immune evasion), and of a direct activity in carcinogenesis through its stimulatory effect on cell proliferation, viability, growth, migration, invasiveness, and metastasis. Figure 2 reports the main biological processes that play a role in tumor development and for which an effect of PA28αβ has been demonstrated.

### 2.1. Role of PA28αβ in the Anti-Tumor Immune Response

The main biological function of PA28αβ has been associated with an increase in the efficiency of MHC class I antigen presentation [110,115]. This is based on some substantial considerations, namely that both PA28α and PA28β are strongly induced by γ-interferon and that the PA28αβ heterocomplex is highly expressed constitutively in immunological tissues and organs (e.g., thymus and spleen), and in professional antigen presenting cells [116,117,118,119]. However, the exact molecular mechanisms through which PA28αβ improves MHC class I presentation have not yet been clearly elucidated [74]. Expression of PA28α alone or in association with PA28β enhances the presentation of some, but not all, class I epitopes, while reduction of PA28αβ levels decreases the proteasomal generation of certain antigenic peptides [120,121,122,123,124,125,126,127,128]. Furthermore, after the immunoproteasome subunit β5i, PA28αβ appears to be the second most relevant UPS element for generation of class I epitopes [124], although its role is more important for certain MHC alleles than for others [129]. Consequently, alterations in the expression and function of PA28αβ that reduce production of MHC class I epitopes from tumor antigens are considered a potential mechanism through which cancer cells evade the control of the immune system [130,131,132,133].

The expression levels of PA28α and β have been analyzed in surgically collected human tumor samples and in tumor cell lines of different origin, in the context of a broader evaluation of the expression of other components of the MHC class I antigen processing machinery (APM) (i.e., immunoproteasome catalytic β-subunits, TAP1/2, aminopeptidases, calnexin, calreticulin, tapasin) and HLA class I antigens. Collectively, the results of these analyses do not offer a clear and homogeneous picture on the possible role of PA28αβ in the emergence of tumor immune escape phenotypes. This is likely due to the great variety in the biological samples examined, which differ in terms of the tumor type and TNM stage, and to the inhomogeneity of the methods used to evaluate the expression of PA28αβ (e.g., WB, HIC, RT-PCR). In fact, in the few cases of tumor lesions analyzed to date, PA28αβ has been reported to be down-regulated, compared to matched normal tissues, in human cervical carcinoma [134], up-regulated in feline injection site sarcoma [135] and in endometrial cancer [136], and unchanged in human gastric cancer [137]. Moreover, PA28α was reported to be highly expressed in human primary and metastatic prostate cancer and to represent a potential target for antibody-mediated therapeutic strategies [138]. Similarly, increased levels of PA28α vs. adjacent healthy tissue have recently been described in rectal cancer [139].

More data are available concerning PA28αβ expression in human or mouse primary and immortalized tumor cell lines of different origin. In this case, the general picture that seems to emerge is that of a reduction in the level of PA28αβ expression (along with several other antigen-presentation related genes) in cancer cells, although this is often true only for some of the tumor clones analyzed [140,141,142,143,144,145], and unaltered levels have also been reported [146,147,148]. In this regard, it is however worth noting that several of these studies have also clearly shown that the expression PA28αβ can be restored to high values by stimulation with γ-interferon [140,142,144,145,147], as presumably occurs normally in an extremely pro-inflammatory context in which most of tumors develop in vivo. Few studies have addressed the molecular mechanisms behind the reduction of PA28αβ levels observed in some cancers, which has been attributed in one case to increased expression of Hsp27 [149] and in another to the proto oncogene HER-2/neu [150].

A potential role of PA28αβ in affecting the efficacy of experimental tumor vaccination protocols has also been evaluated. In this regard, it has been reported that PA28αβ is necessary for the development of effective anti-tumor immunity induced by some vaccines [151,152,153], but not by others [154]. Furthermore, PA28αβ, unlike other components of the MHC class I APM, does not play a key function in inducing a class I immune response that is capable of preventing the spread of lung metastases in a mouse model of fibrosarcoma [155].

The exact role of PA28αβ in the processing of class I tumor-associated antigens has been investigated in only a limited number of cases. Specifically, the proteasome activator has been reported to enhance the generation of a class I epitope derived from p53 [156], one from tyrosine kinase JAK1 [157] and two from tyrosinase-related protein TRP2 [121,123]. Conversely, in a subsequent study PA28αβ was shown to prevent, both in vitro and in cellulo, the correct proteasomal processing of an immunodominant epitope from melanoma antigen A (MART-1) [158], which appears to be in line with previous studies indicating that immunoproteasomes inefficiently generate class I epitopes from self-antigens [159]. Importantly, a recent study comprehensively evaluated the effect of pharmacological stimulation of PA28αβ activity on the MHC class I immunopeptidome presented by multiple myeloma (MM) cells [160]. Using a molecule identified through high-throughput screening that increases the expression levels of the α and β subunits of PA28 and the β5i subunit of the immunoproteasome and promotes the specific association of PA28αβ with the immunoproteasome, a strong boost in the number and diversity of epitopes presented on MHC class I molecules was observed. As a result, the cytotoxic activity of allogeneic and autologous T lymphocytes against multiple myeloma cell lines and patient bone marrow-derived CD138^+^ cells was effectively stimulated. On the other hand, recent research has also demonstrated a very negligible role of PA28αβ in the generation of several MHC class I epitopes (and more generally in supporting the anti-viral immune response), while highlighting its important function in inducing CD8^+^ T cell-mediated graft rejection, although the underlying molecular mechanism is still unclear. Crucially, the overall results of this study strongly suggest that PA28αβ may play a subtle function in generating only a limited number of self-class I epitopes [161]. Of note, this conclusion is in line with in vitro degradation studies showing that, by binding with the 20S particle, PA28αβ enhances the specific generation of only a limited number of individual peptides that are capable of fitting in the MHC-I groove, whereas in general it favors release of fragments too small to serve as class I epitopes [18]. Thus, it appears clear that the role played by PA28αβ in the processing of MHC class I tumor epitopes likely varies depending on the specific type of cancer and antigen and the prevalent form of proteasome present in tumor cells (immuno or constitutive proteasome). Further studies are, therefore, necessary to identify the exact contexts in which PA28αβ plays a prevalent negative role in the generation of tumor epitopes (and therefore from a therapeutic point of view its inhibition would be advantageous) and those in which it favors their correct and efficient processing (and in this case it would be useful to stimulate its activity).

### 2.2. Non-Immunological Role of PA28αβ in Neoplastic Transformation and Tumor Development

A number of studies have focused on the direct role of PA28αβ in carcinogenesis, beyond its function in modulating the effectiveness of cell-mediated immunity. The results of these studies, performed using both tumor cell lines and mouse models, have made it possible to reveal that PA28αβ fulfills specific functions in the process of neoplastic transformation and tumor spread, whose molecular mechanisms are, however, still poorly understood in detail. PA28α was found to be overexpressed in oral cell carcinoma (OSCC), and high levels were associated with recurrence and increased mortality [162]. Importantly, down-regulation of PA28α expression in OSCC cell lines reduced cell viability, proliferation, colony formation, migration, and invasiveness without, however, any detectable effect on cell apoptosis and angiogenesis. Furthermore, PA28α silencing strongly reduced the formation and growth of OSCC xenografts in BALB/C nude mice [162]. Similarly, high levels of PA28α in MM cell lines have been observed and its expression in plasma cells was found to increase with disease progression [163]. Unexpectedly, however, PA28α silencing protected MM cell lines from proteasome inhibitor-induced cell growth repression and apoptosis, while PA28α up-regulation achieved the opposite effect. The interpretation of these data is particularly challenging since in this experimental system the silencing of PA28α also leads to an increase in the expression of the constitutive proteasome catalytic subunits (β1, β2, β5) and PA28γ along with a decrease in those of the immunoproteasome (β1i, β2i, β5i). Importantly, the down-regulation of PA28α in MM was also found to cause a concomitant decrease in steady state levels of PA28β as a consequence of a non-transcriptional mechanism based on stabilization of the protein already described in other cell types [111,164]. Collectively, however, these data demonstrate that PA28α plays a key role in regulating cell growth and proliferation in MM.

Mice deficient in both the PA28α and β genes were generated 20 years ago and were apparently healthy, had no obvious apparent anatomical abnormalities, and lived to at least 1 year [123]. Disappointingly, however, there are scarce data in the literature on the effect of PA28α on apoptosis, and more generally on cell viability, in the context of other pathologies or unphysiological conditions, which would likely be useful to shed light on its role also in neoplastic diseases. In this regard, over-expression of PA28α was reported to slow down retinal degeneration and improve photoreceptor survival in an experimental mouse model of retinal degeneration [165] and to reduce cardiomyocytes apoptosis induced by oxidative stress [111] in a mouse model of diabetic cardiomyopathy [166]. In analogy, PA28αβ was shown to protect MEF cells from a decrease in cell growth induced by oxidative stress [167]. Conversely, in right ventricular hypertrophy and RV failure, over-expression of PA28α was shown to attenuate cardiac hypertrophy and cardiomyopathy and improve survival, although apoptosis of myocytes and non-myocytes was not affected [168]. Moreover, a correlation between enhanced levels of PA28α mRNA expression and a high incidence of neuron apoptosis in rat mesencephalon has been reported [169].

The effect of modulating PA28β levels has also been investigated in different types of cancers. In a gastric adenocarcinoma cell line, over-expression of PA28β resulted in inhibition of cell growth and proliferation, together with its tumorigenicity in nude mice [170]. Similarly, up-regulation of PA28β in two other gastric adenocarcinoma cell lines caused an attenuation of their invasive ability in vitro, while its down-regulation led to the opposite effect [171]. Moreover, in an esophageal squamous cell carcinoma cell line over-expression of PA28β was shown to reduce proliferation, growth, and colony formation and to cause cell-cycle alterations with a clear increase of cells in G1 phase [172]. Finally, in three breast cancer cell lines, silencing of PA28α and of PA28β individually, or of PA28α and β together, had scarce effects on cellular growth rates (only one in three lines showed any difference), while a clear impairment of cell motility and invasive ability was evident for all three knockdown conditions [173]. When injected in nude mice, these stable PA28α and β silencing clones generated much less pulmonary nodules than wild-type control cells, thus implicating a role of PA28αβ in promoting metastasis of breast cancer cells.

Collectively, the data summarized up to this point indicate incontrovertibly that the α and β subunits of PA28 play a role in neoplastic transformation and tumor development, although their specific effect appears to be at least in part dependent on the type of tumor and the experimental conditions. Moreover, the molecular mechanisms through which PA28αβ affect the process of carcinogenesis are not very well understood. Limited data suggests that this activity can be attributed to an effect on the proteasomal degradation of specific protein substrates. For example, increased expression of PA28β has been proposed to induce an accelerated degradation of chloride intracellular channel 1 (CLIC1), a cancer metastasis-associated protein, whose steady state levels show a positive correlation with the invasive and metastatic abilities of gastric cancer cells [171]. Furthermore, PA28αβ was suggested to promote breast cancer cell invasion and metastasis by promoting proteasomal degradation of cyclin-dependent kinase 15 (CDK15) [173]. Moreover, a physical interaction between N-α-acetyltransferase 10 protein (Naa10p) and PA28β (and indirectly PA28α) has been reported [174]. Naa10p is known to regulate several pathways related to cancer cell proliferation, metastasis, apoptosis, and autophagy. Through its association with PA28αβ, Naa10p was shown to negatively modulate chymotrypsin-like proteasomal activity, providing a further possible link between the effect of PA28αβ on proteasome degradative properties and biological processes that play a pivotal role in neoplastic transformation. From a molecular point of view, however, a role of PA28αβ in promoting proteasomal degradation of specific pro- or anti-tumor factors must take into account the well-known biochemical properties of this activator. In fact, PA28αβ was originally described as stimulating the degradation exclusively of short peptides by the proteasome, as a consequence of strong enhancement of all three peptidase activities of the 20S core particle [43,44]. On the contrary, the in vitro degradation of full length proteins by the 20S proteasome, regardless of whether they are ubiquitinated, properly folded, or completely denatured, in all cases analyzed is not stimulated by its association with PA28αβ [7,18,43,44,175]. However, a specific function of PA28αβ, predominantly in association with the immunoproteasome in promoting the degradation of oxidized proteins has also been described [111,112,113,167,176]. In this respect, it is worth noting that many of the in vivo studies reporting an increased efficiency of the proteasomal degradative system following the enhanced expression of PA28α generally document a decrease in the levels of polyubiquitinated proteins and an intensified turnover rate of ectopically expressed substrates (e.g., GFP-CL1), whose degradation necessarily requires their prior ubiquitination [111,164,166]. Therefore, although it cannot be completely ruled out that a few specific substrates may be preferentially degraded by a proteasome formed exclusively by the association of 20S with one or two PA28αβ (perhaps with the involvement of some yet to be identified cellular factor, it seems more likely that the increased hydrolysis of full-length proteins observed following PA28αβ up-regulation may be carried out by so-called hybrid proteasomes containing both a PA28αβ activator and a 19S regulatory complex bound at the opposite ends of a 20S particle [177,178,179,180]. These proteasomes, whose cellular levels have been shown to increase following the enhanced expression of PA28αβ [111,164], are able to recognize polyubiquitinated proteins through specific receptors present in 19S particle (subunits RPN1, RPN10 and RPN13) and to carry out denaturation thanks to the ring of the 6 ATPases subunits (RPT1-6) in contact with the 20S α-annulus [2]. The PA28αβ sitting at the other end of the core particle thus might conceivably enhance the proteolytic efficiency of the hybrid proteasome through long distance allosteric modifications, whose existence has recently been unequivocally demonstrated [69,86,89].

## 3. Conclusions and Future Perspectives

Although a primary function in promoting the neoplastic transformation has always been attributed to PA28γ, experimental evidence accumulated over the years show that its paralogue PA28αβ can also play a decisive role in this process. However, as discussed, the exact molecular mechanisms underlying the carcinogenic activities of PA28αβ are less clear. In this regard, further studies are required to clarify the following crucial points.

(1) What is the role of PA28αβ in the processing of tumor antigens and in the generation of the related MHC class I epitopes? In light of the data concerning its activity in the generation of viral class I peptides, it seems conceivable that PA28αβ does not perform a univocal and general function, but rather varies depending on the type of antigen and tumor examined, and on the concomitant state of the other components of the class I antigen processing machinery. From this point of view, both in vitro and in vivo analyses that examine a greater number of tumor antigens belonging to different classes (both tumor-associated and tumor-specific) are needed.

(2) What is the exact function of PA28αβ in protein degradation, and in particular towards denatured and/or oxidized proteins? In this regard, there is some discrepancy between the results of in vitro experiments (obtained with highly purified components) and those in vivo, which leads to the suspicion that there are other factors involved in the PA28αβ-induced stimulation of full-length proteins by proteasomes. The identification of these factors, which could be molecular chaperones as suggested by some studies [181,182,183], remains a priority to understand the role of PA28αβ in carcinogenesis and beyond.

(3) Does PA28αβ perform its cellular functions primarily in association with the ATP-independent 20S core particle alone, or as a component of ATP-dependent hybrid 26S complexes (i.e., 19S-20S-PA28αβ proteasomes)? In the first case, it would most likely constitute a facilitated and more efficient route for proteins into the proteasomal proteolytic chamber. In the second case, it is conceivable that it could only stimulate the enzymatic activity of the proteasomal proteolytic sites, while substrates could access through the 19S particle according to the canonical modalities. A clear and definitive answer to this question would allow to clarify the function of PA28αβ in the degradation of oncogenic and tumor suppressor proteins and therefore its specific role in the development of various tumors.

The answers to these questions would be greatly facilitated by the development of new tools that allow for the specific differentiation of the activity of PA28αβ. In this regard, the identification of highly selective inhibitors of PA28αβ, and possibly of the 20S and 26S proteasomes, is also important.

## Figures and Tables

**Table 1:** Main structural, cellular, and biochemical differences between PA28αβ and PA28γ.

	PA28αβ	PA28γ
Subunit stoichiometry	3α4β or 4α3β	7γ
Central channel diameter (top-base)	20 Å–30 Å	22 Å–32 Å
Homolog specific insert (i.e., loop between α-helices 1 and 2 that constitute the most divergent portion between α, β, and γ monomers) [58]	Shorter	Longer
Subcellular localization	Cytoplasmic and nuclear	Predominantly nuclear (not in nucleolus)
Constitutive expression	Lymphoid cells and immunological organs	Every organ (high levels in brain and spleen)
Effect of interferon-γ and other pro-inflammatory cytokines on protein levels	Up-regulation	None
Evolutionary distribution	Jawed vertebrates (no birds) [56]	Jawless and jawed vertebrates (orthologs in invertebrates and unicellular eukaryotes)
Effect on the enzymatic properties of the 20S proteasome	Stimulation of short peptides hydrolysis	Stimulation of peptides and unfolded proteins hydrolysis
Effect on sizes distribution of peptide products released by 20S	Reduced	Unaffected or only marginally affected
